# Characterization of Green Part of Steel from Metal Injection Molding: An Analysis Using Moldflow

**DOI:** 10.3390/ma16062516

**Published:** 2023-03-22

**Authors:** I Putu Widiantara, Rosy Amalia Kurnia Putri, Da In Han, Warda Bahanan, Eun Hye Lee, Chang Hoon Woo, Jee-Hyun Kang, Jungho Ryu, Young Gun Ko

**Affiliations:** 1 School of Materials Science & Engineering, Yeungnam University, Gyeongsan 38541, Republic of Korea; 2 Kyerim Metal Co., Ltd., Chilgok 39910, Republic of Korea

**Keywords:** green part, metal injection molding, Moldflow, steel

## Abstract

Metal injection molding (MIM) is a quick manufacturing method that produces elaborate and complex items accurately and repeatably. The success of MIM is highly impacted by green part characteristics. This work characterized the green part of steel produced using MIM from feedstock with a powder/binder ratio of 93:7. Several parameters were used, such as dual gates position, injection temperature of ~150 °C, and injection pressure of ~180 MPa. Analysis using Moldflow revealed that the aformentioned parameters were expected to produce a green part with decent value of confidence to fill. However, particular regions exhibited high pressure drop and low-quality prediction, which may lead to the formation of defects. Scanning electron microscopy, as well as three-dimensional examination using X-ray computed tomography, revealed that only small amounts of pores were formed, and critical defects such as crack, surface wrinkle, and binder separation were absent. Hardness analysis revealed that the green part exhibited decent homogeneity. Therefore, the observed results could be useful to establish guidelines for MIM of steel in order to obtain a high quality green part.

## 1. Introduction

Steels, relative to other metals, can be processed easily, utilizing conventional fabrication [1,2]. However, in order to form steels with sophisticated shapes, unconventional ways must be used. Metal injection molding (MIM) is a shape manufacturing process, which combines plastic injection molding with powder metallurgy. MIM permits the manufacturing of small parts with a combination of useful characteristics, such as using inexpensive materials, low-cost production, broad composition selection, and high density with mechanical properties in comparison with wrought materials [1,3,4]. There are four steps in MIM, namely, mixing of metallic powder with binder to form a feedstock, injection of feedstock into the mold resulting in a green part, debinding to remove the binder, and sintering to decrease porosity [5]. The processing parameters, such as the composition of metallic powder and binder to make the feedstock, must be carefully selected, as they will determine the properties during the rest of the process [6,7]. Other parameters, such as size of powder for feedstock, parameter for injection molding, debinding, and sintering, are considered vital in order to obtain final product with minimum to no defects [8,9,10,11]. To obtain the proper parameters for certain materials, especially designed characterization, techniques, together with sound simulation modelling, will be needed. Among the known simulation programs, C-Mold, ProCAST, and Moldflow, are commonly used nowadays to predict the melt flow front, the weld lines’ position, the temperature, and the pressure. Moldflow software was highlighted as the most promising commercial package, since it is statistically in agreement with the experimental results and can simulate multiple processes in one run [12,13,14].

A number of studies on MIM have been carried out where simulation was utilized to assess the process performance. However, for widespread implementation, a number of objectives must be met, such as a significant amount of data that must be collected in order to generalize the modelling result with all those parameters and their variation [15,16]. Current research on titanium with a relatively new version of Moldflow modelling software showed a good agreement between experimentation and simulation [17]. The filling progress of MIM predicted by Moldflow successfully verified the experimental results, with a maximum and minimum error of 9% and 1.5%, respectively. A review on processing of iron using MIM on an industrial scale suggested that, to convince the community to adopt the MIM method, simulations have become a standard requirement needed for quality control consideration [11].

In this study, steel processed by MIM will be investigated by using experimental testing together with simulation analysis using Moldflow. The current study will focus on investigating the green part as a first step to achieve the MIM final product. X-ray computed tomography (XCT) will be used to extract the three-dimensional projection of the interior of the green part for a comprehensive experimental analysis. The experimental and simulation results will be discussed based on the proposed parameters, which include the ratio of metallic powder and binder, the dual gates position, the injection pressure, and the injection temperature. From this study, we hoped to establish guidelines for producing high quality green parts of steel via the MIM method.

## 2. Materials and Methods

### 2.1. Powder and Feedstock

The model was a valve sleeve fcv made from SUS440C with a chemical composition of Fe—1.20%, C—1.00%, Si—1.00%, Mn—1.04%, P—0.03%, S—0.60%, Ni—18.00%, and Cr—0.75% Mo (in wt.%). The mixing temperature was set at 120 °C. The powder binder ratio was 93:7 (mass%).

### 2.2. Metal Injection Molding (MIM)

**Figure 1:**
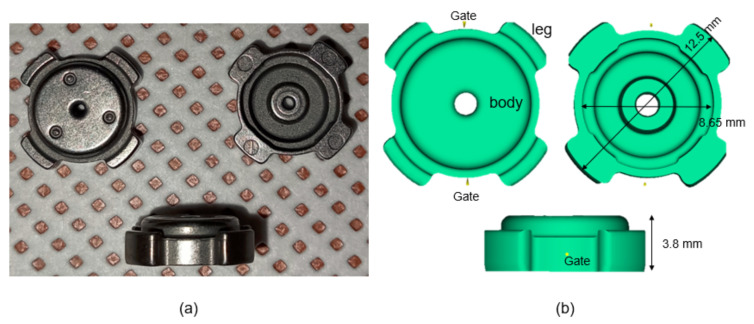
(**a**) Green parts and (**b**) Moldflow model.

The case study was a valve sleeve (Figure 1a) with the main dimension provided in Figure 1b. Two different gates were designed for the present molds process. Feedstock was injected by the plunger of an injection molding machine with an injection temperature of ~150 °C and an injection pressure of ~180 MPa. The feeding system consisted of two gates positioned face-to-face to each other, as shown in Figure 1b. The ratio of powder/binder was 97:3. The quality of MIM is determined to a great extent by the mixture of metallic powder and binder [18].

### 2.3. Moldflow Simulation

The simulation analysis (Figure 1b) was realized using Moldflow plastic insight. The three-dimensional model was generated as a stp format file and then imported into Moldflow software for analysis. The gate location on the green part was determined by using Moldflow software. Injection parameters, such as temperature and pressure, were set as close as that of the real condition. Two gates’ positions were set accordingly. As shown from the Moldflow model in Figure 1b, the green part will, therefore, consist of two regions and will be termed as body and leg regions.

### 2.4. X-ray Computed Tomography (XCT)

For X-ray tomography, the X-ray microscope Xradia 620 Versa (Carl Zeiss, Jena, Germany) was used with a spatial resolution of 0.5 μm, a voltage of approximately 30~160 kV, and maximum output of 25 W.

### 2.5. Hardness Analysis

From the tribology test, stainless steel was known to show optimum properties in density, hardness, and wear rate. Thus, steel via MIM shows a promising future for tribological material [19]. A series of Vickers hardness measurements was carried out on the samples with polished surfaces under the load of 200 g, with a holding time of 10 s.

## 3. Results and Discussion

### 3.1. Moldflow Simulation

**Figure 2:**
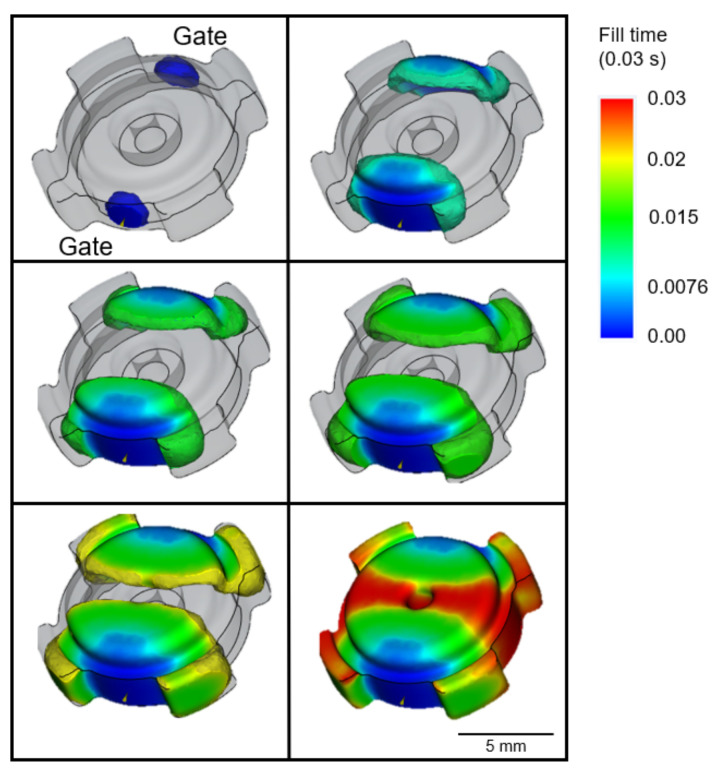
Filling progress.

The filling progression of the MIM with the process parameters mentioned above are predicted by the Moldflow software, as shown in Figure 2. The simultaneous filling from the two gates was achieved so that over-packing along the flow paths can be avoided. Based on the final shape of the green part achieved at very short filling time of ~0.03 s, short shot did not occurred, which was attributed to the appropriate injection pressure (~180 MPa) and temperature (~150 °C) [20]. The utilization of two gates supported the likelihood of part filling in all sections [21]. A previous study revealed that dual gates are preferable, as they imposed very good symmetry in term of metallic powder distribution [22]. Other work utilizing Moldflow on MIM with a dual gate system has been reported elsewhere [23].

**Figure 3:**
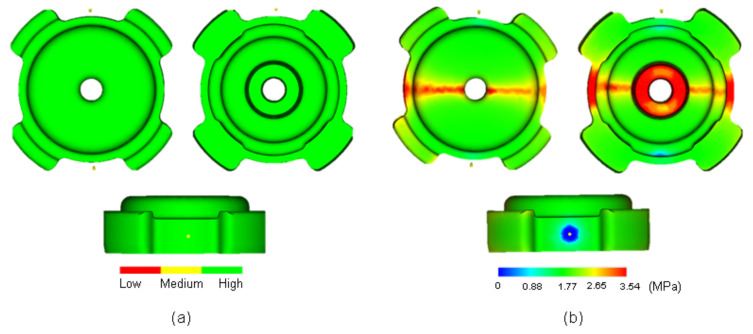
(**a**) Confidence to fill and (**b**) pressure drops.

**Figure 4:**
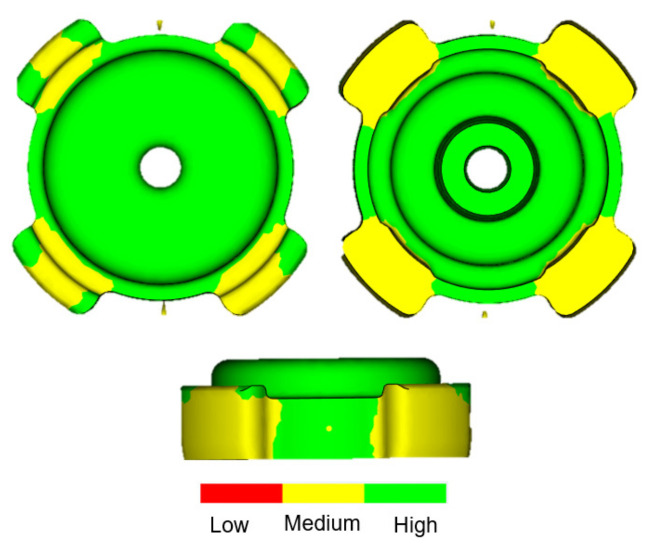
Quality prediction.

Confidence to fill, resulting from Moldflow (Figure 3a), revealed that the entire body of the green part has high value (green color) and no indication of the occurrence of short shot. It was suggested that processing conditions were deemed incorrect if confidence of fill result was low [24]. One of the Moldflow results is pressure drop, as shown in Figure 3b. The color used here indicated the region of highest pressure drop (red) through to the region of lowest pressure drop (blue) [25]. The physical meaning of the high value of the pressure drop may indicate an adversity to fill a particular area of the green part. As shown in Figure 3b, some regions exhibited high values of pressure drop in the body of the green part along the red-colored region (pressure drop outline) where the flow of feedstock from the two gates met. A high value of pressure drop may occur due to several reasons: insufficient injection pressure and temperature, as well as the number of gates used for injection. The latter was expected to be the main reason for the high pressure drop. The pressure drop may lead to a filling problem or short shot, which were already seen to not occur based on the final product and the filling progress result shown above. Another consequence of pressure drop is the low value of confidence to fill. In Moldflow, the value of confidence of fill is derived from pressure drop. In order to improve the value of confidence to fill, the injection pressure must be increased. Interestingly, the confidence of fill for the Moldflow simulation of this study was high, despite the occurrence of a pressure drop in some particular region. Another essential Moldflow result is quality prediction (Figure 4). The availability of this value further confirmed the absence of short shot phenomenon. Low value of quality prediction could mean that the pressure drop exceeded its maximum [26]. However, despite the occurrence of pressure drop along the red-colored region in Figure 3b, quality prediction value on that region was surprisingly high. The leg regions of the green part, on the other hand, exhibited medium value for quality prediction. Unlike confidence to fill, quality prediction is a derivation of the temperature, pressure, and others. Thus, the level of sophistication to interpret this result will be much higher as compared to previously mentioned results. To support the simulation results, the green part will be assessed experimentally using both scanning electron microscopy (SEM) for surface analysis and X-ray computed tomography (XCT) for the interior analysis of the green part.

### 3.2. Surface Observation

**Figure 5:**
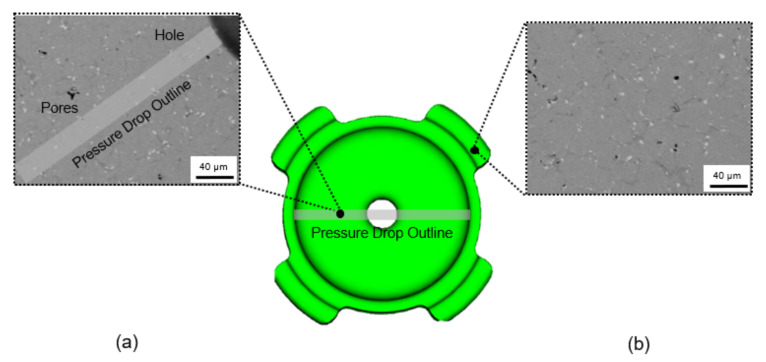
SEM images for (**a**) region with pressure drop and (**b**) low quality prediction.

Around the time when the filling process was nearly completed, surface wrinkle may easily occur when injection parameters were poorly adjusted [27]. At low temperature, feedstock will partially form into semi-solid phases. When the feedstock was imposed with excessive pressure, the semi-solid phase would be deformed, and surface wrinkles would occur at the surface. This was expected to occur when the opposite feedstock met at the middle of the green part along the pressure drop outline, as shown in Figure 3b. SEM observation revealed that no surface wrinkle was observed along the pressure drop outline, as shown in Figure 5a. Therefore, the experimental result was in agreement with the Moldflow simulation, showing that, despite the drop in term of pressure along the pressure drop outline during mold filling, quality prediction in that area was surprisingly high (Figure 4). With similar conditions, the defect in the form of jetting may occur if the gate was improperly designed, causing the feedstock to freeze partially [27]. Since jetting was not detected around the gate of the current sample, which is also in agreement with the simulation showing perfect shape around the gate, the current dual gate positioning in this study was effective in accommodating the movement of feedstock. Other than the mentioned conditions, the current feedstock with a powder/binder ratio of 93:7 was expected to play an important role in giving good flowability [28]. This can be observed clearly, since Moldflow considers the rheological properties of the feedstock. Therefore, the utilization of Moldflow would be favorable for the injection stage and a model with a complex shape [17]. Nevertheless, some small irregular pores were detected, since the surface of the feedstock after solidification was pretty rough, and pores can easily form in these areas near the mold wall.

SEM observation revealed that a small fraction of pores was formed on the surface of the leg area around the region with medium value of quality prediction (Figure 5b). For most cases, the outer region would take a longer time to fill, and cracks tend to form in the contact area between the solidified feedstock in the front and the following with higher temperature [29]. Moreover, insufficient injection pressure caused the feedstock to not integrate well during solidification, and this led to defect formation. This was in agreement with the Moldflow filling progress in Figure 2, where the leg area was completed at the final moment of the filling process (shown as red region). Since the two gates were located near the leg region, the occurrence of semi solidification of feedstock would be minimized. This once again would provide a very small chance for the feedstock to solidify and to make contact with the non-solidified counterpart, which inhibited the formation of cracks.

### 3.3. Three-Dimensional Observation

**Figure 6:**
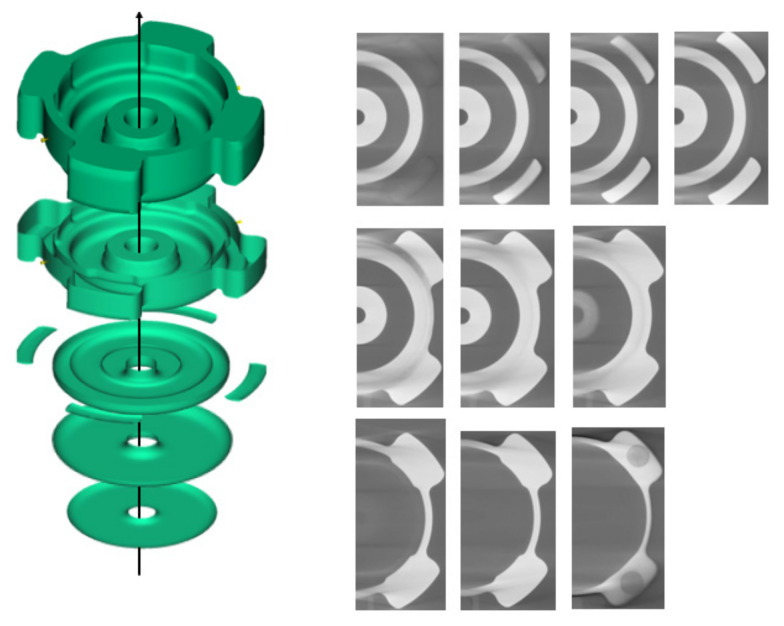
XCT images along the axis (black arrow).

The profiling of the interior of the green part will be performed using XCT. This method is deemed superior to other methods, such as dimensional measurements and the hydrostatic Archimedean technique, which require a large amount of work and can be tedious [30]. Other traditional techniques required a process of sample cutting, mounting, and polishing, which are therefore destructive. Thus, XCT can be considered as an alternative to characterize the interior of the green part in a quick and non-destructive manner [31]. One critical defect in the MIM process is binder separation. Binder separation may occur due to shearing of feedstock, and separation of powder from the binder may occur, which result in packing inhomogeneities [32]. Previous simulation of the literature reported that the binder separation between powder and binder was a phenomenon which happened during mold filling with high shear rate of different densities of powder and binder [33]. Unlike the other defects, binder separation occurred when injection was applied at a high temperature. Thus, the selected temperature for this process must not be too high. One of the indicators for the occurrence of binder separation is the formation of cracks in the interior of the green part, separating two regions with different powder–binder composition. A series of three-dimensional observation images obtained using XCT are presented in Figure 6 along the axis shown in the image. From the images, no trace of cracks was found in either the body or the leg of the green part. A series of three-dimensional observation images obtained using XCT are presented in Figure 6 along the axis shown in the image. From the images, no trace of cracks was found in either the interior of the body or the leg region of the green part.

It was reported previously that binder separation can be avoided if the feedstock exhibits good flowability, which depends on viscosity or powder–binder ratio of the mixture up to certain critical concentration [34]. In this experimental result, as shown in Figure 3b, despite the occurrence of pressure drop along the pressure drop outline where the two flows of feedstock from the two gates met, the value of the coincidence to fill the whole area was surprisingly high in all regions. This suggested that the feedstock exhibited good flowability due to selection of injection temperature and pressure. In addition, the flowability can be considered as a fingerprint for a good ratio of metallic powder to binder, which means that the current powder ratio can be considered as optimum. The current powder ratio of 93:7 fulfills the requirement, such that feedstock formulations should contain about 2–5% extra, exceeding the necessities to fill the interstices between particles [35].

**Figure 7:**
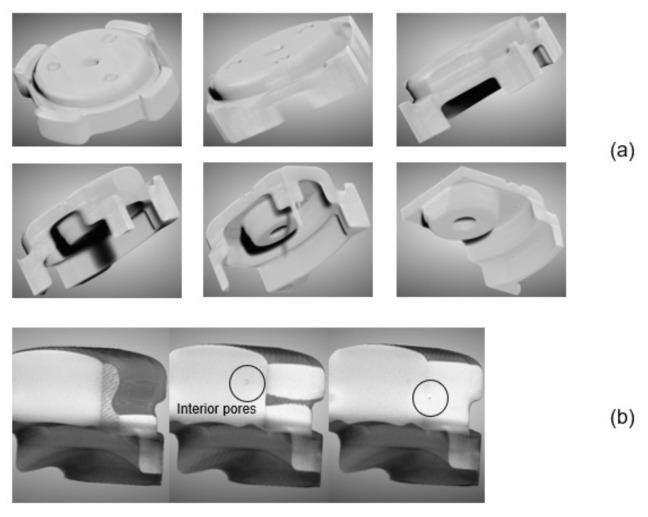
XCT images of (**a**) the whole body and (**b**) the leg of the green part.

Some pores were found to form in the interior of the green part for the case of the body and the leg region, as shown in Figure 7. The utilization of two gates may have resulted in feedstock rapid movement around all the sections, and the vent of the mold may be blocked, and some air was left in the mold [29]. Consequently, the residual air leads to pore formation.

### 3.4. Hardness

Binder separation is a phenomenon, which is frequently observed in powder injection molding and happens during the molding process, which can induce inhomogeneities, especially in term of densities of the green parts. In mild cases, such inhomogeneities might originate from the pores [36,37], while, in severe cases, inhomogeneities can be associated with severe binder separation, which can be seen from a localized hardness value.

**Figure 8:**
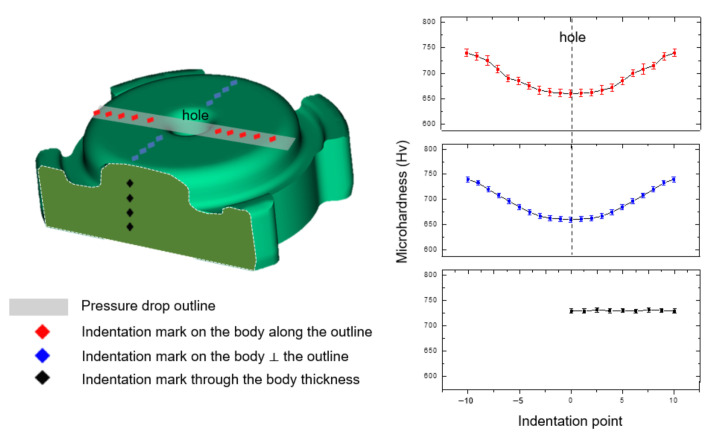
Vickers hardness analysis along the three traces. Indentation along the pressure drop outline (red), perpendicular to pressure drop outline (blue), and perpendicular to previous two traces (black).

In line with the Moldflow result of pressure drop, Vickers hardness indentation was carried out along the pressure drop outline that may be subject to pressure drop phenomenon (Figure 8). For comparison, a series of indentations was carried out perpendicular to the pressure drop outline (represented with the color of blue). In addition, hardness test through the thickness of the green part was carried out (black). Hardness test results show that the hardness value along the pressure drop outline increased with increasing distance from the hole. The result along pressure drop outline was pretty close to those measured perpendicular to it. For hardness test through the body thickness, a different tendency was observed, where the hardness variation was negligible. There was no localized hardness found in all observation, signifying that binder separation did not happen.

## 4. Conclusions

In this work, the green part of steel fabricated via MIM was characterized and analysed using Moldflow was carried out. The green part was prepared via MIM by injecting feedstock with a powder/binder ratio of 93:7 through two gates at temperature of 150 °C and pressure of 180 MPa. The Moldflow results showed that, despite the high confidence to fill, a dual gate injection caused a pressure drop, as well as low quality prediction in the leg parts. Scanning electron microscopy, as well as three-dimensional examination using XCT, was utilized to observe the structure. It was revealed that surface wrinkle, cracks, and binder separation were not observed, and only a very small amount of pores was detected around the pressure drop outline and the leg part. Hardness measurement using the Vickers indenter revealed the homogeneities in term of densities and that binder separation was not happening. Thus, MIM with the current injection parameters was successful to mold the homogeneous samples without critical defects, such as cracks, surface wrinkles, and binder separation. The results in this study can help green part designers, as well as MIM manufactures, to determine important parameters in order to improve the quality of the green part. Further work to relate the current results to distortion and shrinkage effects observed during debinding and sintering process will be needed.

## Data Availability

The data presented in this study are contained within the article.
